# Better Control of Holder Pasteurization Results in Higher Retention of Human Milk Lactoferrin, IgA, and Lysozyme

**DOI:** 10.3389/fped.2018.00381

**Published:** 2018-12-03

**Authors:** Rachel Buffin, Stéphane Hays, Jocelyne Drai, Marie-Nathalie Sarda, Jean-Charles Picaud

**Affiliations:** ^1^Neonatology Department, Croix-Rousse Hospital, Lyon, France; ^2^Régional Rhône Alpes Auvergne Human Milk Bank, Hôpital de la Croix-Rousse, Lyon, France; ^3^Biochemistry Laboratory, Lyon-sud Hospital, Pierre-Bénite, France; ^4^Immunology Laboratory, Lyon-sud Hospital, Pierre-Bénite, France; ^5^CarMen Unit, INSERM U1060, INRA U197, Claude Bernard University, Pierre-Bénite, France

**Keywords:** human milk bank, donor human milk, quality control, milk processing, immunity

## Abstract

**Background:** Holder pasteurization is commonly used in milk banks. We previously reported that the pattern of temperature and time may be different according to the pasteurizer used.

**Aim:** The aim of our study was to assess the variances in pasteurization using two different devices: a standard pasteurizer (Past STD) and an optimized pasteurizer (Past OPTI).

**Methods:** Immunoglobulin A (IgA), lactoferrin (LF), and lysozyme (LZ) content were assessed before and after pasteurization of 24 donor human milk samples. The impact of the pasteurization device was evaluated by testing 50- to 200-mL samples.

**Results:** Mean temperature and duration of the plateau were 1.5°C lower and 11 min shorter, respectively, with Past OPTI vs. Past STD. The loss of IgA, LF, and LZ was 17.6, 5.6, and 9.8% lower, respectively, with Past OPTI than with Past STD.

**Conclusions:** Accurate control of temperature enabled better preservation of IgA, LF, and LZ in donor milk. Holder pasteurization should be optimized, and new techniques proposed to treat donor milk should be compared with Holder pasteurization performed with a well-controlled device under realistic conditions.

## Introduction

Human milk (HM) is the gold standard for very-low-birth-weight infant nutrition. Its antimicrobial and immunomodulatory components, such as lactoferrin (LF), immunoglobulin A (IgA), or lysozyme (LZ) compensate for the deficit in neonatal immune system and contribute to the prevention of sepsis in these vulnerable infants (1–5). The mother's milk is the first choice, but when unavailable, donor HM from a milk bank is the best alternative (6). The safety of donor HM is a main concern of HM banks and is achieved by pasteurization. The reference method used worldwide is Holder pasteurization, which consists of heating the milk at a low temperature (62.5°C) for a long duration (30 min). Holder pasteurization partially destroys some HM components (1). Most relevant studies have been performed *in vitro* with very small samples of HM, with discrepancies in the results (1, 7). These discrepancies could be related to differences in pasteurizer performance, which has not been evaluated and described in most studies, as it is assumed that all pasteurizers have the same performance, which is not the case (8).

Indeed, Czank et al. reported that the impact of heat treatment on HM properties depends on temperature. Between 40 and 57°C, immune components were stable but dramatically decreased above 58°C, lactoferrin being the most affected (9). Moreover, we previously reported that pasteurization temperature was different depending on the type of pasteurizer. Time and temperature during the pasteurization process were inconsistent when using an air-ventilated pasteurizer. Not all bottles were exposed to the same temperature for the same duration, resulting in heterogeneous pasteurization. With a water pasteurizer, pasteurization was more homogenous than with an air-ventilated pasteurizer. Furthermore, we reported that an optimized pasteurizer produced better results than a non-controlled one (8). Optimization was achieved by a precise adjustment of the machine to comply with recommendations (1, 8). It could be helpful to preserve bioactive components of HM (5). The benefits of HM are well-documented (6) and most of them are related to the immune component (2, 3) The aim of the study was to assess LF, IgA, and LZ content of donor HM before and after Holder pasteurization using two water pasteurizers: a standard device (Past STD) and an optimized device (Past OPTI).

## Materials and methods

This study was performed at the regional HM bank (Lactarium Auvergne Rhone-Alpes, LARA) at the Croix-Rousse University Hospital in Lyon, France.

Two devices from the same manufacturer (HSC, Décines, France) were used for this study: a standard device (PAS 10000 first version) and an optimized device (PAS 10002). In both devices, bottles were partially immerged and agitated continuously to ensure temperature homogenization. Cooling process differed: Past STD cooled the milk with ambient tap water, while Past OPTI cooled the milk down to 4°C with a tank of refrigerated water. Past STD was an older pasteurizer compliant with standard temperature regulation. Past OPTI was designed with a new regulation system offering a lower temperature and shorter duration of plateau during pasteurization cycle. Prior to study we characterized each pasteurizer by recording the temperature during a pasteurization cycle, using external probes as previously recommended (1, 8). Past STD did not adhere to the following criteria previously proposed (8) (such as the mean temperature between 62.5 and 64°C and plateau duration between 30 and 35 min. By contrast, the temperature pattern of Past OPTI were in agreement with these criteria.

Donors provided written informed consent for the use of their milk for this research purpose. The milk used for the study was frozen donor milk that was collected for research use and could not be used for premature infants because of contraindication according to the French national guidelines (herbal intake, transfusion, >4 months of storage, or smoking) (10).

### Study design

**Figure 1:**
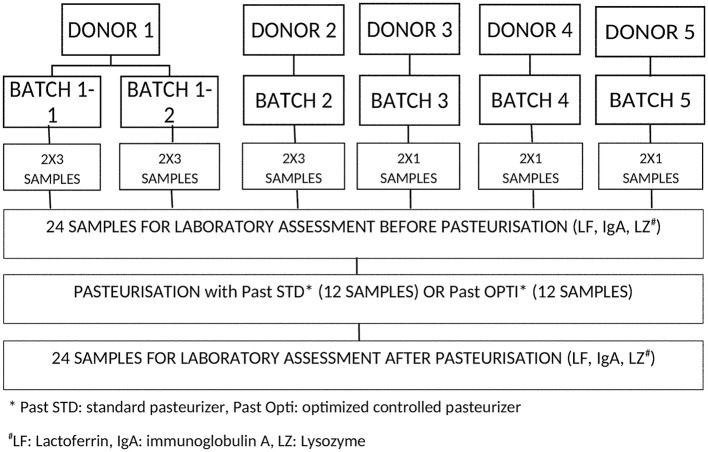
Design of the study and distribution of samples tested with the two pasteurizers: Past STD represents the, standard pasteurizer, while Past OPTI represents the new pasteurizer with better regulation. Each donor's milk was divided in order to be assessed in each device. Each sample was assessed for lactoferrin, IgA, and lysozyme before and after pasteurization.

The milk from each donor was thawed, poured into an Erlenmeyer flask and homogenized by manual stirring. The milk was divided into 24 single-use polypropylene bottles (Beldico SA, Marche-en-Famenne, Belgium) (Figure 1). A sample of 10 mL was collected from each bottle before pasteurization.

**Figure 2:**
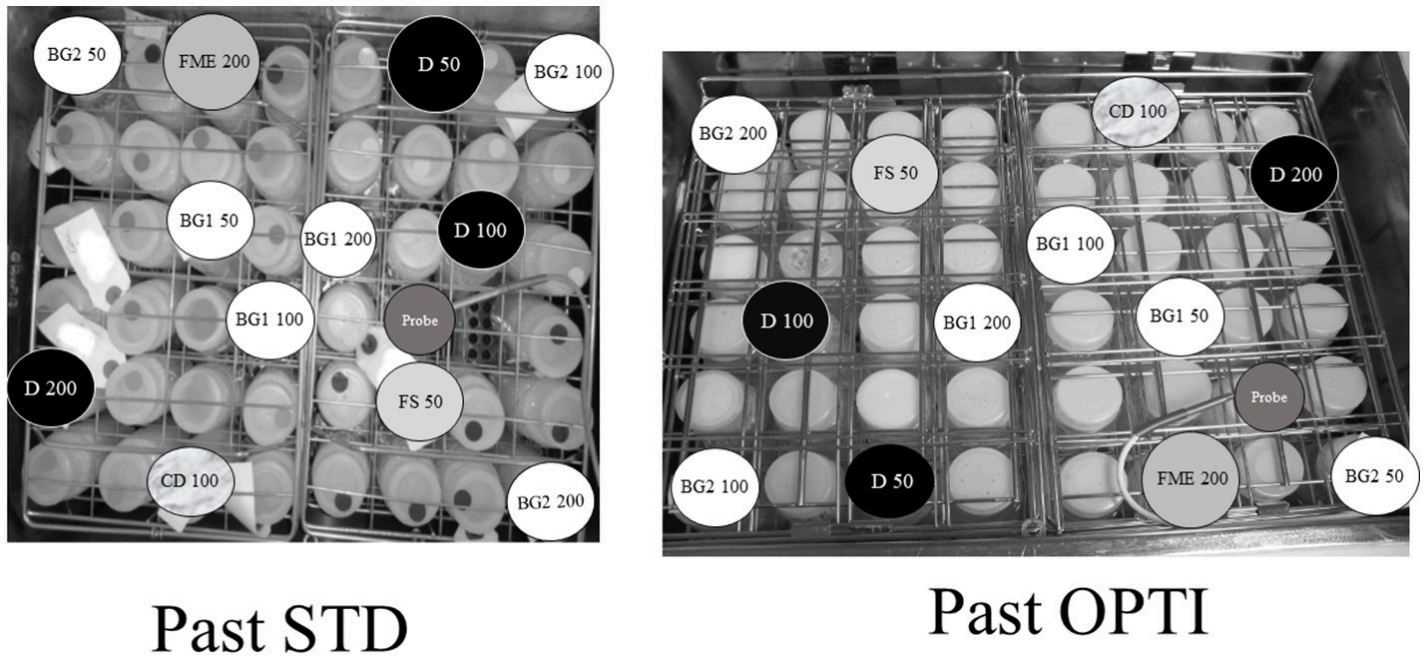
Distribution of the samples in the Past STD and Past OPTI pasteurizers. Capital letters represent the identification of the donor. The volume in each bottle is indicated.

The bottles (50–200 ml) were then similarly distributed within each pasteurizer (Past STD and Past OPTI) and subjected to a routine pasteurization cycle (Figure 2). A second 10-mL sample was collected from each bottle after pasteurization.

All samples were anonymously labeled, for blind analysis frozen at −21°C and carried to the laboratory in an icebox. Blinded assessment of LF, IgA, and LZ before and after pasteurization was performed at the biochemistry and immunology laboratories in Lyon-Sud Hospital using enzyme-linked immunosorbent assay.

As the range of LF and IgA values was broad, the coefficients of variation were calculated with a low and high value of each component. The range of values was narrower for LZ, and therefore the coefficient of variation was calculated based on a single value. Because of technical problems, LZ content was not assessed in three samples.

### Statistical analysis

We calculated the mean and standard deviation of the LF, IgA, and LZ content before pasteurization. We evaluated the impact of pasteurization on LF, IgA, and LZ concentrations by expressing the results as the difference between the concentration before and after pasteurization and finally as the percentage of retention after pasteurization. This was calculated for both pasteurizers. Differences in absolute value and percentage of pre-treatment value were compared by a Wilcoxon matched sample test. The threshold of significance was set at 0.05. The software used for analysis was SPSS® version 19 (IBM SPSS Statistics, Boigny-sur-Bionne, France).

## Results

The characteristics of the pasteurization cycles of both pasteurizers were different (Table 1). The mean plateau temperature and duration were 1.5°C lower and 11 min shorter, respectively, with Past OPTI than with Past STD (Table 1).

**Table 1:** Characteristics of pattern of holder pasteurization of Past STD and Past OPTI.

	**Past STD**	**Past OPTI**
Mean plateau temperature (°C)	64.4	< 62.9
Min plateau temperature (°C)	62.7	62.6
Maximum plateau temperature (°C)	64.8	62.9
Mean plateau duration over 62.5°C (min)	42	31

The coefficient of variation for assessment of LF and IgA was 6.3 and 4.6% for lower values (mean values tested: 106 and 735 mg/L) and 11.4 and 3.6% for higher values (mean values tested: 713 and 1,591 mg/L), respectively. The coefficient of variation for LZ assessment was 7.4% (mean value tested: 143 mg/L).

**Figure 3:**
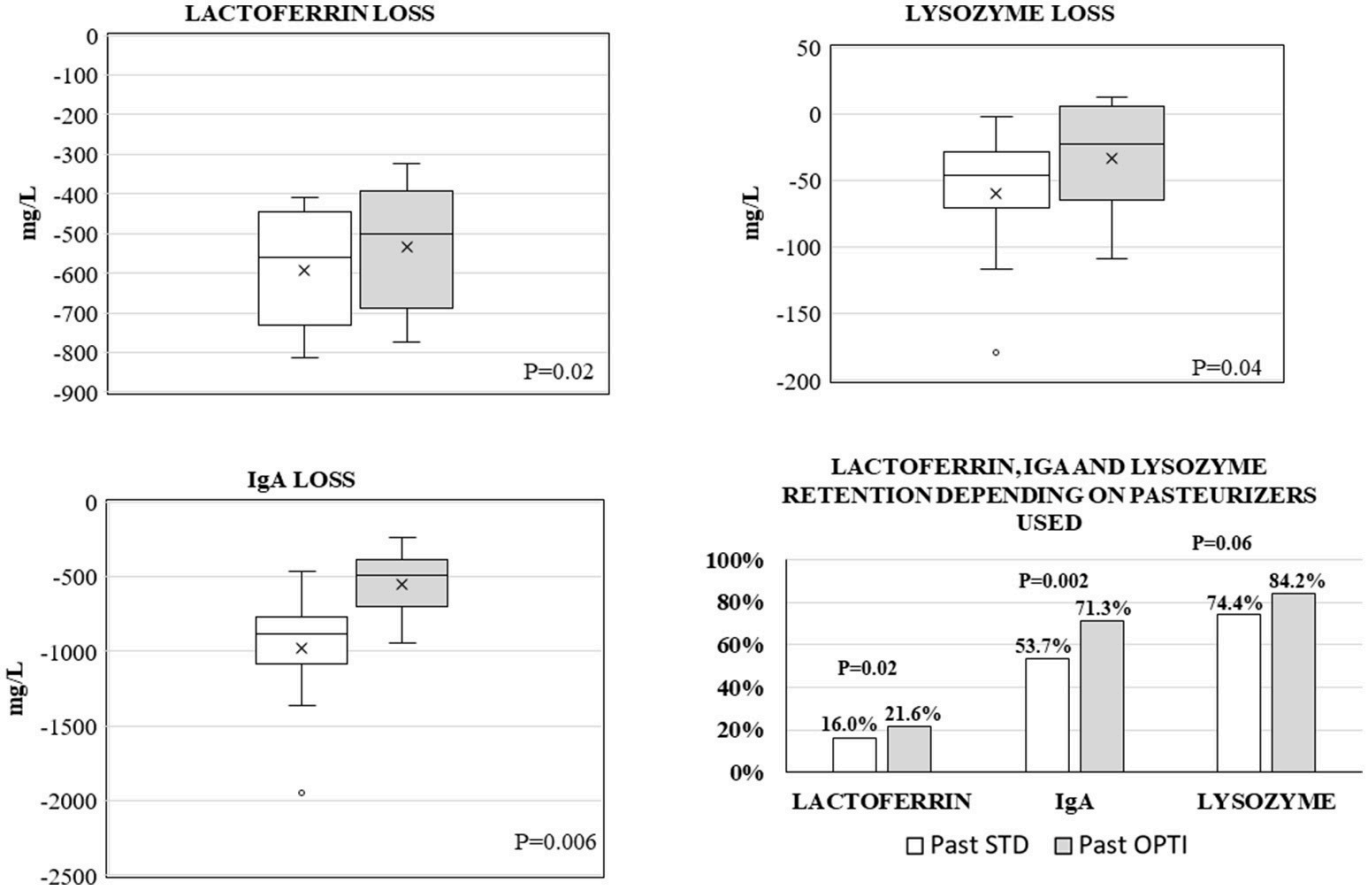
Schematic representation of loss of lactoferrin, IgA, and lysozyme after Past STD (white) and Past OPTI (gray) pasteurization expressed by mean (x) and median (–) in boxplots. The values of preservation are also presented as percentages depending on the pasteurizer used.

Median (min, max) values before pasteurization were 631 (465, 915) mg/L for LF, 1,976 (1,103, 2,528) mg/L for IgA, and 195 (135, 357) mg/L for LZ. Reduction in LF, IgA, and LZ was greater when using Past STD compared with Past OPTI, with a median reduction of −559.5 (−813, −410) mg/L vs. −499.5 (−772, −322) mg/L (*p* = 0.02) for LF, −887.5 (−1,947, −465) mg/L vs. −494.5 (−948, −239) mg/L (*p* = 0.006) for IgA, and −46.5 (179, −2) mg/L vs. −24 (−109, 13) mg/L (*p* = 0.037) for LZ, respectively (Figure 3). Retention of immune components after pasteurization was ~20% for LF, 60% for IgA, and 80% for LZ (Figure 3). Retention was significantly higher with Past OPTI than with Past STD: 21.6 vs. 16% for LF, 71.3 vs. 53.7% for IgA, and 84.2 vs. 74.4% for LZ, which represented a gain of +5.6, +17.8, and +9.8 points for LF, IgA, and LZ, respectively (Figure 3).

## Discussion

In this study, we observed that an optimized pasteurization better preserved the immune components compared with a standard pasteurization owing to strict control of HM exposure to heat.

The effect of exposure to different temperatures during the Holder pasteurization process is a remaining matter of concern. Czank et al. described the differences between three types of pasteurizers (bottle immersion (sterifeed), Holding chamber (saurin industries), and an experimental one designed for the study allowing precises' measures of temperature (Carag AC). The impact of the different pasteurizers was not drastic with respect to LF and IgA but was of concern for LZ, in favor of the experimental pasteurizer (9). However, the temperature patterns were not available for these devices, and the composition of treated HM was different. A strength of our study is that we analyzed precisely the temperature pattern of both pasteurizers and we used the same milk for measurements before and after pasteurization to avoid skewing of data due to variations in milk composition (11, 12). Meredith-Dennis et al. also showed differences in LF, IgA, and LZ contents assessed in HM treated with different methods (Holder pasteurization, vat technique or retort sterilization). Indeed, LF, IgA, and LZ—concentrations were greater after Holder pasteurization than after other methods. However, as the samples of HM were different and randomly selected, it was difficult to disentangle effects related to the selection of HM and to the difference in pasteurization methods (11).

In our study, we observed that better temperature control had a statistically significant impact on the retention of three major immune components in HM. We used these components as markers because their effects are well-known, and clinical benefits may be achieved from increasing their concentration in donor HM (13). These markers are commonly used, allowing comparison with levels in other studies (1). In fact, the concentrations of immune components measured in our study were within the range of previously reported values (12, 14–17). Although we did not assess the concentration of other components, we expect that it might also be impacted by the improvement of temperature control.

In recent reviews, the retention after pasteurization ranged from 10 to 65% for LF, 38 to 80% for IgA, and 31 to 80% for LZ, but most articles did not specify the pattern of pasteurization of the devices (1, 7), which could be responsible for these discrepancies (1). Furthermore, nearly all previous studies were performed with very small samples of HM (1). A strength of our study is that it was carried out under conditions closest to routine working conditions of HM banks In such a context we observed that excepted for LF, the retention rates measured in our study were in the upper range of retention reported in the literature (53 and 71% of IgA, 74 and 84% of LZ) (7). It suggests that the impact of Holder pasteurization could be much less than previously published, under the condition that it is a good quality pasteurization (18, 19).

The reduction in immune components following the Holder pasteurization is well-known (1, 7). Therefore, new techniques have been proposed such as high pressure processing, high temperature-short time pasteurization or ultraviolet-C (20–22). The feasibility of their routine use in HM banks is still to be evaluated. Furthermore, the impact on HM bioactive components should be assessed in conditions as close as possible to HM bank, i.e., in large enough milk samples (50–200 mL) and using pasteurizers (not laboratory devices). Finally, it should be compared to the reference method, i.e., Holder pasteurization, performed with devices using a stringent control of temperature during the whole pasteurization cycle (8).

A limitation of our study is that samples were frozen before reaching the laboratory. Indeed, Akinbi et al. reported that freezing induced supplementary loss of immune components (12). However, it is unlikely that the freezing influenced the comparison between pasteurizers, as all HM samples were handled similarly. Furthermore, it suggests that the percentage of immune components preserved after Holder pasteurization was underestimated.

Another limitation is that the only biological parameters measured were the concentrations of three major immune components. Although our study was not designed to investigate the relationship between retention of immune components and clinical evolution, it is well-known that beneficiary effects of HM on health of preterm infants are due in part to its composition (2, 3, 23). HM helps reduce the occurrence of nosocomial bacteremia and the risk of late-onset sepsis in preterm infants (2, 23, 24) It can be assumed that the efficacy of HM is proportional to the quantity of immune components. Therefore, assessing the concentrations of these components may be considered useful until further clinical studies are able to clearly identify benefits related to improvements in pasteurization process.

In conclusion, our results suggest that better control of temperature during Holder pasteurization can improve preservation of LF, IgA, and LZ. Holder pasteurization is used worldwide in HM banks, because it offers the best compromise between efficiency and feasibility. Therefore, it is essential to use only pasteurizers that underwent a stringent control of temperature pattern commercially available. When new techniques are proposed for donor HM treatment, they should be compared under realistic conditions with a well-controlled pasteurizer.

## Data availability statement

The raw data supporting the conclusions of this manuscript will be made available by the authors, without undue reservation, to any qualified researcher.

## Author contributions

RB and J-CP designed, directed the project wrote the paper with input from all authors. SH, RB, and J-CP analyzed the data. M-NS and JD performed the measurements and analyzed data.

### Conflict of interest statement

The authors declare that the research was conducted in the absence of any commercial or financial relationships that could be construed as a potential conflict of interest.

## Abbreviations

HM: Human milk
IgA: Immunoglobulin A
LF: Lactoferrin
LZ: Lysozyme
Past OPTI: Optimized pasteurizer
Past STD: Standard pasteurizer.
